# Mercury Exposure Levels in Children with Dental Amalgam Fillings

**DOI:** 10.5005/jp-journals-10005-1261

**Published:** 2015-02-09

**Authors:** Indu Miriam Varkey, Rajmohan Shetty, Amitha Hegde

**Affiliations:** Former Postgraduate Student, Department of Pedodontics and Preventive Dentistry, AB Shetty Memorial Institute of Dental Sciences, Mangalore Karnataka, India; Professor, Department of Pedodontics and Preventive Dentistry, AB Shetty Memorial Institute of Dental Sciences, Mangalore Karnataka, India; Professor and Head, Department of Pedodontics and Preventive Dentistry, AB Shetty Memorial Institute of Dental Sciences, Mangalore Karnataka, India

**Keywords:** Amalgam, Mercury levels, Hair sample, Urine sample, Toxicity.

## Abstract

**Objectives:** Mercury combined with other metals to form solid amalgams has long been used in reconstructive dentistry but its use has been controversial since at least the middle of the 19th century. The exposure and body burden of mercury reviews have consistently stated that there is a deficiency of adequate epidemiological studies addressing this issue. Fish and dental amalgam are two major sources of human exposure to organic (MeHg) and inorganic Hg respectively.

**Materials and methods:** A total of 150 subjects aged between 9 and 14 years were divided into two groups of 75 subjects each depending on their diet, i.e. seafood or nonseafood consuming. Each category was subdivided into three groups based on number of restorations. Scalp hair and urine samples were collected at baseline and 3 months later to assess the organic and inorganic levels of mercury respectively by atomic absorption spectrophotometer (AAS).

**Results:** The mean values of urinary mercury (inorganic mercury) in the group of children with restorations were 1.5915 μg/l as compared to 0.0130 μg/l in the groups with no amalgam restorations (p < 0.001) (Wilcoxon sign rank test and paired t-test). The hair mercury levels (organic mercury) varied signi-ficantly between the fsh-eating group and nonfsh-eating group, the average values being 1.03 μg/l and 0.84 μg/l respectively (p < 0.001) (Mann-Whitney U-test and paired t-test).

**Conclusion and significance:** The notion about the mercury being released from the amalgam restorations as a sole exposure source needs to be put to a rest, as environmental factors collectively overpower the exposure levels from restorations alone.

**How to cite this article:** Varkey IM, Shetty R, Hegde A. Mercury Exposure Levels in Children with Dental Amalgam Fillings. Int J Clin Pediatr Dent 2014;7(3):180-185.

## INTRODUCTION

The use of mercury and its combinations with other metals in dentistry dates back to centuries ago. Dental amalgam contains about 50% mercury, with the remainder mainly silver. Although alternative dental materials are increasingly available for posterior fllings, amalgam has advantages that maintain its popularity as a flling material. These include relatively low cost, increased durability, and less sensitivity to clinical technique than other materials.^1^ The use of mercury in dentistry has been controversial since at least the middle of the 19th century. This controversy has intensified lately, because of techniques showing mercury to be continuously released from dental amalgam fllings.^2^

Mercury is a metallic element that occurs naturally in the environment. There are three primary categories of mercury and its compounds: elemental mercury, which may occur in both liquid and gaseous states, inorganic mercury compounds and organic mercury compounds (MeHg).

MeHg is present as a result of the methylation of inorganic Hg by microorganisms, usually present in sediments. It undergoes a remarkable biomagnification process and accumulates in the fish muscle tissues of long-lived predatory species, such as sharks in ocean waters.^3^

Elemental mercury is the main form of mercury released into the air as a vapor by natural processes. Elemental mercury can be oxidized by the hydrogen peroxide-catalase pathway in the body to its inorganic divalent form. Elemental mercury (Hg^0^) emitted to the atmosphere is converted to soluble forms, deposited into soil and water, and methylated to methyl mercury (MeHg). Fish and dental amalgam are two major sources of human exposure to organic (MeHg) and inorganic mercury respectively.

The exposure from dental amalgam occurs mainly by inhalation of elemental mercury evaporating from the flling.^4^ Mercury vapor absorption occurs through the lungs, with about 80% rapidly entering the blood stream. Following distribution by blood circulation, mercury can enter and remain in certain tissues like the central nervous system and the kidneys for longer periods of time. The following factors have been listed as variables affecting the release of mercury from, amalgam restorations: number of teeth, number of surfaces, baseline mercury release, magnification factors, such as eating and tooth brushing, oral breathing habits, nose-mouth breathing ratio, inspiration-expiration ratio, swallowing, inhalation absorption, ingestion absorption, body weight.^5^

Some mercury species, such as methyl mercury accumulate at higher concentrations in hair, making them relatively easy to measure. Mercury remains stable for long periods in hair, making it easy to transport and store. Mercury also has a longer half life in hair, hence, useful for evaluating exposures that occurred months earlier.^6^

No conformational studies of the past provide consistent results of mercury toxicity,^7,8^ hence, this study was carried out to investigate the organic and inorganic levels of mercury among the pediatric age group.

## MATERIALS AND METHODS

One hundred and ffity subjects either males or females, of the age group ranging from 9 to 14 years, belonging to both fish eating and nonfsh eating categories and living in the South Kanara district, Karnataka, India, were included in the study. Informed consent was obtained from each of the subjects. They were healthy subjects with no known prior or existing restorations. They were included if fully erupted permanent maxillary and mandibular first molars on both right and left sides were present with at least one being carious. The carious lesions being treated belonged to the class I genre of Black's classification which is moderately deep. Subjects who were uncooperative or those with underlying physician diagnosed psychological, behavioral, neurological, immunosuppressive or renal disease were excluded.

They were divided into two equal groups of 75 subjects each depending on their diet, i.e sea food consuming or nonseafood consuming subjects. The subjects belonging to the ‘sea food consuming’ category were those who have been eating sea food thrice weekly for at least the past 2 months. The ‘nonseafood consuming’ category consisted of pure vegetarians. Once the subjects were included into the study group, their diet was restricted to three servings of sea food per week.

The 75 subjects in the ‘sea food consuming’ category were subdivided into three groups as follows:

- * Group 1:* Subjects requiring 1 to 2 restorations (n = 25)
- * Group 2:* Subjects requiring 3 to 4 restorations (n = 25)
- * Group 3:* Control group with no restorations (n = 25)

Further, the subjects under the ‘nonseafood consuming’ category were subdivided into three groups as follows:

- * Group 4:* Subjects requiring 1 to 2 restorations (n = 25)
- * Group 5:* Subjects requiring 3 to 4 restorations (n = 25)
- * Group 6:* Control group with no restorations (n = 25) The dental materials used in this trial were universally accepted tooth flling materials (Dentsply). All dental treatments met the existing standards of care.

Scalp hair samples were collected from each of the subjects belonging to all groups to assess the organic levels of mercury. A single strand of hair was collected on the day of examination for baseline values and then 3 months later and was subjected to the atomic absorption spectrophotometer (AAS).^9^

Urine samples were collected from the control group subjects and before the start of any restorative procedure in the study group subjects for baseline values and 3 months post flling in all the study groups and control groups to assess the inorganic levels of mercury. The urine sample (~10 ml, morning mid stream sample) collected from each subject of study and control groups was subjected to the cold vapor technique together with atomic absorption spectrophotometer (CVAAS) for analysis.^10^ The samples were digested before analysis with nitric acid to a homogenous solution. This would release bound mercury as Hg^2+^ from protein sulfur complexes.

Participants and dentists could not be blinded to treatment assignment, but all those collecting outcome data or analyzing the specimens at the laboratory were blinded to the child's treatment assignments.

Comparisons were made between the amalgam treatment group and the control group with and without seafood consumption in terms of the urinary mercury concentration (for inorganic and elemental mercury levels)^11^ and scalp hair mercury concentration (for organic mercury levels).^12^

## STATISTICAL ANALYSIS

The various observations were subjected to statistical analysis as follows:

1. Variations in mercury levels before and after restorations–Wilcoxon sign rank test.
2. Variations within each group at baseline and 3 months later in urine and hair samples paired t-test.
3. Comparison of mercury levels between fish eaters and nonfsh eaters Mann-Whitney U-test.
4. Comparison of mercury levels in children having restorations and control groups with no restorations-Mann-Whitney U-test.
5. Comparison of mercury levels between boys and girls Mann-Whitney U-test.

## RESULTS

Table 1 indicates the variation in mercury levels in urine and hair samples between baseline and 3 months later analyzed using the Wilcoxon sign rank test. The increased levels of mercury were found to be statistically significant (p < 0.001) in both hair and urine samples after 3 months from baseline values.

Table 2 shows the variation within each group at baseline and 3 months later in urine samples analyzed using the paired t-test. The values increa sed significantly (p < 0.001) in all the study groups, i.e. the groups with amalgam restorations, whereas the slight increase seen in control groups were not significant (p = 0.007). Group 4 showed an increase from 0.21 μg/l to 1.62 μg/l, and group 5 showed a higher elevation of mercury levels from 0.21 μg/l to 2.10 μg/l, showing a correlation between increased levels of mercury to increased surfaces of restorations.

Table 3 shows the variations in mercury levels of hair between each of the six subgroups in this study evaluated using the paired t-test. The values increa sed significantly in all the fish eating groups (p < 0.001), whereas the levels did not increase significantly in the nonfsh eating groups (p = 0.001).

Table 4 compares the differences in levels of mercury in hair and urine samples between the fish eaters and nonfsh eaters, using the Mann-Whitney U-test. The difference in mercury levels in urine of fish eaters was 1.11 μg/l and of nonfsh eaters was 1.26 μg/l. This difference was not statistically significant (p = 0.181). The difference in mercury levels in hair of the fish eaters was 1.03 μg/l and 0.08 μg/l in nonfsh eaters, the difference between the two groups being statistically significant (p < 0.001). Table 5 compares the differences in levels of mercury in hair and urine samples between the study groups (with restorations) and the control groups (without restorations) done using the Mann-Whitney U-test. The difference in mercury levels in urine of the study groups (i.e. with restoration) was 1.59 μg/l and of the control groups (i.e. without restoration) was 0.01 μg/l. This difference was statistically significant (p < 0.001). The difference in mercury levels in hair of the study groups (i.e. with restoration) was 0.32 μg/l and 0.43 μg/l in the control groups (i.e. without restoration ), the difference between the two groups not being statistically significant (p = 0.333).

**Table 1:** **Table 1:** Variation in mercury levels in urine and hair samples between baseline and 3 months later (μg/l) – Wilcoxon sign rank test

		*N*				*Minimum*		*Maximum*		*Percentiles*						*Mean rank*		*Z*		*Asymp. sig. (2-tailed)*	
		*Valid*		*Missing*						*25*		*Median*		*75*							
Urine baseline		150		0		0.0190		0.6720		0.12800		0.21450		0.325250		24.42		–10.075		<0.001	
Urine 3 M		150		0		0.0410		2.7730		0.27250		1.4015		1.93000		79.94					
Hair baseline		150		0		0.0140		1.9720		0.25475		0.74050		1.00850		49.83		–10.063		<0.001	
Hair 3 M		150		0		0.0290		4.5600		0.35075		1.2220		2.16750		76.57					

**Table 2:** **Table 2:** Variation within each group at baseline and, 3 months later, in urine samples (μg/1) – paired t-test

				*Valid*		*Min.*		*Max.*		*25*		*Median*		*75*		*Mean rank*		*Z*		*p-value*	
Fish eating with 1/2 restorations (group 1)		Urine baseline		25		0.019		0.672		0.128		0.314		0.43		0.00		–4.372^b^		<0.001	
		Urine 3 M		25		1.009		2.458		1.243		1.523		1.707		13.00					
Fish eating with 3 or more restorations (group 2)		Urine baseline		25		0.125		0.567		0.191		0.325		0.4645		0.00		–4.372^b^		<0.001	
		Urine 3 M		25		1.224		2.682		1.6315		2.089		2.3905		13.00					
Nonfsh eating with 1/2 restorations (group 4)		Urine baseline		25		0.021		0.623		0.1275		0.21		0.249		0.00		–4.372^b^		<0.001	
		Urine 3 M		25		1.008		2.512		1.225		1.625		1.971		13.00					
Nonfsh eating with 3 or more restorations (group 5)		Urine baseline		25		0.026		0.526		0.14		0.214		0.351		0.00		–4.372^b^		<0.001	
		Urine 3 M		25		1.052		2.773		1.876		2.108		2.438		13.00					
Fish eating control group (group 3)		Urine baseline		25		0.052		0.381		0.094		0.171		0.274		18.00		–1.952^b^		0.051	
		Urine 3 M		25		0.041		0.393		0.126		0.186		0.2745		11.75					
Nonfsh eating control group (group 6)		Urine baseline		25		0.026		0.415		0.098		0.167		0.2655		9.14		–2.651^b^		0.007	
		Urine 3 M		25		0.08		0.476		0.127		0.206		0.2625		14.50					

^b^Positive rank

## DISCUSSION

Dental amalgams, commonly known as ‘silver fllings,’ contain mercury, silver, tin, copper and zinc.^13,14^ Liquid elemental mercury (Hg) when added to the other ingredients produce a mass that is moldable enough to be forced into the prepared cavity. Manual pressure is used to squeeze out the excess of Hg. Curing occurs in about a day with the fnal mass containing 45 to 50% Hg by weight.^15^

Dental amalgams have long been believed to contribute little to the body burden of mercury. This is because the elemental form of mercury is rapidly consumed in the setting reaction of the restoration. But, research now shows that amalgam is not en tirely chemically stable after curing. In contrast to earlier studies, recent evidence suggests that amalgam in the oral environment constantly releases small quantities of cytotoxic corrosion products and Hg vapor.^16,17^ The Hg vapor levels are greatly in creased by mildly abrasive action, such as chewing gum and brushing, and ingestion of hot beverages.^18^ The current point of controversy is whether or not the levels released are great enough to be hazardous to the health of the patient.

No large studies have been completed that examine the effects of mercury exposure from dental amalgam fllings in children. This study was carried out among children ranging from 9 to 14 years. These young children are particularly vulnerable to the effects of mercury because their brains are still developing, and greater surface area in the lungs relative to their body weight causing increased inhalation. Also, elemental mercury is heavier than air and higher concentrations may be seen at lower levels near the child's breathing zone.^19^

**Table 3:** **Table 3:** Variation within each group at baseline and, 3 months later, in hair samples (μg/l) – paired t-test

				*N*		*Min.*		*Max.*		*Percentiles*						*Mean rank*		*Z*		*p-value*	
				*Valid*						*25*		*Median*		*75*							
Fish eating with 1/2 restorations (Group 1)		Hair baseline		25		0.714		1.647		0.83		0.902		1.3765		0.00		–4.373^b^		<0.001	
		Hair 3 M		25		1.212		2.301		1.374		1.428		2.0123		13.00					
Fish eating with 3 or more restorations (Group 2)		Hair baseline		25		0.712		1.972		0.92		1.032		1.8275		0.00		–4.373^b^		<0.001	
		Hair 3 M		25		1.701		3.06		1.255		1.357		2.677		13.00					
Nonfsh eating with 1/2 restorations (Group 4)		Hair baseline		25		0.021		1.325		0.1035		0.174		0.379		11.00		–3.285^b^		0.001	
		Hair 3 M		25		0.029		1.532		0.1475		0.221		0.5685		12.27					
Nonfsh eating with 3 or more restorations (Group 5)		Hair baseline		25		0.11		0.916		0.2145		0.314		0.48		23.50		–3.108^b^		0.001	
		Hair 3 M		25		0.1019		1.006		0.311		0.424		0.6685		12.09					
Fish eating control group (Group 3)		Hair baseline		25		0.1042		1.676		0.759		0.881		0.982		0.00		–4.372^b^		<0.001	
		Hair 3 M		25		0.42		1.701		0.82		1.065		1.491		13.00					
Nonfsh eating control group (Group 6)		Hair baseline		25		0.014		1.116		0.136		0.256		0.35		17.00		–3.115^b^		0.001	
		Hair 3 M		25		0.043		1.025		0.2595		0.332		0.444		12.83					

^b^Positive rank

**Table 4:** **Table 4:** Comparison of difference in the mercury levels between fish eaters and nonfsh eaters (μg/l) – Mann-Whitney U-test

				*Valid*		*Min.*		*Max.*		*25 percentile*		*Median*		*75 percentile*		*Mean rank*		*Mann-Whitney U-test*		*Z*		*Asymp. sig. (2-tailed)*	
Differences in urine levels		Fish eaters		75		–0.05		2.44		0.0150		1.1140		1.5900		70.75		2456.500		–1.338		0.181	
		Nonfsh eaters		75		–0.08		2.51		0.0620		1.2630		1.7960		80.25							
Differences in hair levels		Fish eaters		75		0.19		3.35		0.5510		1.0310		1.4887		112.15		64.000		–10.331		<0.001	
		Nonfsh eaters		75		–0.53		0.52		0.350		0.0840		0.1560		38.85							

**Table 5:** **Table 5:** Comparison of difference in the mercury levels in the study groups (with restorations) and control groups (without restorations) (μg/l) –Mann-Whitney U-test

				*N*		*Min.*		*Max.*		*Percentiles*						*Mean ranks*		*Mann-Whitney U-test*		*Z*		*Asymp. sig. (2-tailed)*	
				Valid						*25*		*Median*		*75*									
Differences in urine levels		Restoration present		100		0.40		2.51		1.186		1.5915		1.845		100.50		0.000		–9.967		<0.001	
		Restoration absent		50		–0.08		0.16		0.0003		0.0130		0.0468		25.50							
Differences in hair levels		Restoration present		100		–0.53		3.35		0.0890		0.3260		0.8653		73.07		2257.00		–0.969		0.333	
		Restoration absent		50		–0.90		2.78		0.0690		0.4395		1.451		80.36							

Mercury is a naturally occurring element and exists in three forms: organic, inorganic and elemental^5^ and this study analyses all three exposure forms in children.

## Organic Mercury Level Assessment

Organic/methylmercury which is discarded by industries into the water bodies concentrates in tissues of fish and other sea creatures and moves up the food chain. Fish and marine mammals are the dominant sources, contributing up to 70 to 90% of the total mercury. Larger the fsh, more the concentration of mercury in them. The intake of mercury depends not only on the level of mercury in fish but also the amount consumed. In lieu of the above, fish eating and a nonfsh eating population was selected for comparisons in this study.

Sample collected to assess organic mercury was hair^6^ mainly because mercury has a longer half life in hair and remains relatively stable. In our study, children of the age group 9 to 14 years were chosen confirming no hair treatments to have been done. An occipital hair sample was collected at baseline and, 3 months later, half life of MeHg being around 70 days.

The difference in organic mercury levels in hair samples in our study varied significantly between the fish eating group and nonfsh eating group, the average values being 1.03 μg/l and 0.084 μg/l respectively (p < 0.001). This is in accordance with studies by Salehi et al,^9^ Fakour et al^12^ and Kruzikova et al^20^ who showed the increased concentrations of hair mercury due to seafood consumption. The levels were significantly increased in all the fish eating groups irrespective of the presence or absence of restorations, thus, stating the increase to be from the organic mercury only.

Intake of fish and fish products, averaged over months or weeks, results in an average daily absorption of methy-lmercury variously estimated to be between 2 and 4.7 μg mercury as quoted in literature by Levy M et al^21^ and Suzuk T^22^ in studies done in children, which is in accordance with our study.

The FDA (USA) quotes the maximum allowable concentration of methylmercury to be no more than 1 ppm (1 mg/l) or alternatively they state that a safe intake would be 0.1 μg/kg body weight.^23^ In India, studies done by Ramamurthy (1979) and Bhattacharya and Sarkar (1996), give max permissible limits as 0.5 ppm.^24^ In our study, the minimum and maximum values observed in the fish eating groups were 0.19 μg/l and 3.3 μg/l which is well within the permissible limits for organic levels of mercury in children.^21,22^

## Inorganic/Elemental Mercury

Dental fllings made with mercury amalgam can be a source of human exposure to elemental mercury vapors for many population. Amalgam surfaces release mercury vapor into the mouth and lung, depending upon the number of amalgam fllings and other factors, the estimated average daily absorption of mercury vapor from dental fllings varies between 3 and 17 μg mercury.^24^ Thus, amalgam restoration groups were used as study groups in this study to measure exposure levels as other sources of exposure are highly variable and would not be standardized.

The presence of mercury in urine^25^ generally represents recent exposure to inorganic and/or elemental mercury, and collection is noninvasive. However, inorganic mercury can accumulate in the kidney and slowly get excreted through the urine, thus, also capable of representing exposures to elemental mercury and/or inorganic mercury that occurred sometime in the past.^26,27^ Nicolae A^28^ and Doddes^5^ also state that the most common way to measure mercury exposure is through urine samples, since its fairly easy to collect these samples. Hence, in our study, midmorning samples of urine were collected at baseline and, 3 months later, half life being around 66 days.

The mercury levels in urine increased significantly in all the groups with amalgam restorations from baseline values to a 3-month follow-up period, irrespective of the consumption of seafood. The mean values of urinary mercury in the group of children with restorations were 1.59 μg/l as compared to 0.01 μg/l in the groups with no amalgam restorations (p < 0.001). A proportional increase in urinary mercury levels has been observed with an increase in number of restorations in our study, adhering to the principles of Olsson and Bergman.^29^

The findings thus demonstrate a strong positive association between urinary mercury concentration and number of amalgam surfaces as seen in others studies by Guzzi G,^30^ Woods JS et al^10^ and Xibiao Ye.^31^

Our study is also in agreement with other studies wherein the levels of mercury in urine increased subsequently after amalgam restorations, specifically in children. The New England trial shows median value of 1.5 μg/l ± 1.2,^10^ and Levy et al^21^ showed that, in children aged 4 to 8 years old, children with amalgam fllings (1.412 microg Hg/g) had significantly higher urinary Hg levels than children without amalgams (0.436 microg Hg/g).

Studies on exposed humans do not provide sufficient information to derive acceptable intakes for inorganic mercury compounds; therefore, based on no adverse effects and lowest adverse effects in medium- and long-term animal experiments, ATSDR and IPCS derived a guidance value of 0.2 μg/kg body weight per day for inorganic mercury compounds. The values obtained in our study stays well clear off the maximum permissible limits.

Notably, we observed a constant but quantifable urinary mercury excretion among children in this study who did not receive amalgam restorations. This most likely represents the systemic uptake of mercury from food, air, or other environmental sources like industries, broken instruments, medications, etc.

The observations in this study imply that the level of mercury exposure from all sources including amalgam restorations did not exceed the capacity for elimination via the urinary excretion in these subjects.

The New England trial^31^ did a follow-up for 7 years in children with amalgam restorations and revealed that the inorganic levels of mercury in urine peaked after 3 years and reduced to nil after 7 years. This has been an exclusive longitudinal study done in children and, as per their findings, we could also expect the inorganic levels to come down to baseline values after a few years. Thus, inorganic levels of mercury does not seem to pose a threat as much as organic levels observed in hair which remains fairly constant. Thus, in a coastal area like the South Kanara region in Karnataka, India, where the present study was undertaken, the residents who consume fish on a regular basis could probably be at a higher risk of organic toxicity than an inorganic one. Hence, should amalgam restorations be done in a subject who consumes fish on a regular basis still remains questionable, due to inadequate long-term evaluations of individual mercury levels. Thus, longitudinal studies in the same group of children needs to be carried out to evaluate variations in exposure levels with time.
